# A Randomized Study of a Government Cash Supplement on Cognitive Function among Adults in Malawi

**DOI:** 10.1002/alz70860_103350

**Published:** 2025-12-23

**Authors:** Allison E Aiello, Youngjoon Bae, Farizah Rob, Rebecca C Stebbins, Maxton G Tsoka, Alan A Cohen, Adina Zeki Al Hazzouri, Daniel W. Belsky, Sudhanshu Handa

**Affiliations:** ^1^ Robert N. Butler Columbia Aging Center, Department of Epidemiology, Mailman School of Public Health, Columbia University, New York, NY, USA; ^2^ Robert N. Butler Columbia Aging Center, Columbia University, New York, NY, USA; ^3^ University of Malawi, Zomba, Zomba, Malawi; ^4^ Columbia University Mailman School of Public Health, New York, NY, USA; ^5^ Mailman School of Public Health, Columbia University, New York, NY, USA; ^6^ Butler Columbia Aging Center, Columbia University, New York, NY, USA; ^7^ University of North Carolina‐ Chapel Hill, Chapel Hill, NY, USA

## Abstract

**Background:**

People with higher incomes and more wealth live longer, healthier lives and are less likely to develop dementia. However, whether government programs that supplement income can protect health and prevent dementia is unknown. To test the hypothesis that income supplementation can contribute to dementia prevention, we collected neurocognitive performance data from older adults enrolled in a randomized controlled trial of a cash transfer intervention similar to universal basic income, entitled the Malawi Social Cash Transfer Program (SCTP).

**Method:**

In 2013, the Malawi SCTP was part of a government cash supplement program and randomized 3,531 households to early entry (treatment, 47.5%) or delayed entry in 2016 (control, 52.5%). Cash transfers were scaled by household size and represented ∼20% of pre‐program average household consumption (e.g., $8/month for a family of four), disbursed bimonthly and continued across the follow up period. At follow up in 2021, we administered word‐recall, digit‐span, and Raven's‐matrix tests to 89% of the original participants (*n* = 3,160, 90% women) and computed composite neurocognitive performance scores. Data were available for analysis from 3,030 older adults (avg. age 54). We conducted an intention‐to‐treat analysis using linear regression with adjustment for baseline residential area, age, and sex. We clustered standard errors by village. Sensitivity analyses considered effects by sex and for individual neurocognitive tests. Analyses were conducted with the Stata and R software.

**Result:**

At follow‐up, participants were an average age of 58 years. Participants in households randomized to the early‐entry treatment group had higher neurocognitive test scores as compared with delayed‐entry‐household controls (for a composite cognitive z‐score across tests, b=0.07, 95% CI: 0.01, 0.13). Sensitivity analyses revealed effects were somewhat larger for women than men and for digit‐span‐backwards as compared with the other tests.

**Conclusion:**

A government cash supplement program significantly improved neurocognitive‐test performance in older adults in Malawi. Findings suggest that universal basic income programs in resource‐limited settings could reduce cognitive impairment and ultimately dementia risk.

